# Supplementary material for: PrescDRL: deep reinforcement learning for herbal prescription planning in treatment of chronic diseases
Source: Chin Med. 2024 Oct 16;19:144. doi: 10.1186/s13020-024-01005-w (PMC11481742; doi:10.1186/s13020-024-01005-w)
Supplement: Supplementary file 1 [file 13020_2024_1005_MOESM1_ESM.pdf]

Supplementary Materials for PrescDRL: Deep reinforcement learning for herbal prescription planning in treatment of chronic diseases

1. Case study

In this section, we provide three examples of treatment process comparison given by doctors and PrescDRL. In the first example (Table S1), the algorithm performed better than the doctor. In the second example (Table S2), the doctor performed better than the algorithm, and in the third example (Table S3), both the doctor and the algorithm performed better.

Table S1 A diagnosis and treatment example given by doctors and PrescDRL.

Type	Treatment number	Symptom of patients	TP	Prescription	Symptom score
Doctor	1	咳嗽,舌苔薄,汗多,脉缓,胃热,舌苔黄,呕吐,恶心,脉沉,口干渴,呕吐,反酸	93	麸炒枳实,浙贝母,柏子仁,炒苏子,五味子,苦杏仁,太子参,莱菔子,大黄,麦冬,法半夏,炒决明子	12
	2	口苦,舌苔薄,肛门灼热,脉缓,胃热,屁多,舌苔黄,脉沉,小便尿热,口干渴	7	黄芪,浙贝母,柏子仁,海螵蛸,炒苏子,五味子,苦杏仁,制何首乌,太子参,莱菔子,大黄,麦冬,黄连,制吴茱萸,炒决明子	10
	3	口苦,失眠,头晕,舌苔黄,大便干,食少,心慌,口干渴,脉象弦,全身麻木	63	麸炒枳实,砂仁,酸枣仁,柏子仁,五味子,龙骨,牡蛎,太子参,珍珠母,大黄,麦冬,黄连,法半夏,茯苓	10
PrescDRL	1	咳嗽,舌苔薄,汗多,脉缓,胃热,舌苔黄,呕吐,恶心,脉沉,口干渴,呕吐,反酸	93	麸炒枳实,白芍,酸枣仁,浙贝母,柏子仁,五味子,龙骨,牡蛎,太子参,珍珠母,大黄,麦冬,生地黄,法半夏,炒决明子	12
	2	胃胀,舌苔白,胃热,小便尿频,大便干,口干渴,脉细	93	麸炒枳实,黄芪,砂仁,酸枣仁,丹参,柏子仁,五味子,檀香,太子参,大黄,麦冬,炒决明子	7
	3	胃胀,舌苔白,胃热,大便干,脉细	93	麸炒枳实,黄芪,砂仁,酸枣仁,丹参,柏子仁,五味子,太子参,麦冬,炒决明子	5

- TP denotes the treatment plan.
- The English translations for the symptom names mentioned in the case are as follows: 咳嗽(cough), 舌苔薄(thin coated tongue), 汗多(hidrosis), 脉缓(slow pulse), 胃热(stomach heat), 舌苔黄(yellow tongue coating), 呕吐(emesis), 恶心(nausea), 脉沉(deep pulse), 口干渴(thirsty mouth), 反酸(sour regurgitation), 口苦(bitter taste), 肛门灼热(burning pain in anus), 屁多(Lot of farts), 小便尿热(hot urination), 失眠(insomnia), 头晕(dizziness), 大便干(dry stool), 食少(reduced appetite), 心慌(palpitation), 脉象弦(pulse string), 全身麻木(general numbness), 胃胀(gastric distension), 舌苔白(white tongue coating), 小便尿频(frequent urination), 脉细(thin pulse).
- The English translations for the Chinese herbal names mentioned in the case are as follows: 浙贝母(bulb of Thunberg Fritillary), 生地黄(Fresh Rehmannia root), 黄连(Rhizome of Chinese Goldthread), 砂仁(Villous Amomum fruit), 海螵蛸(Cuttlebone), 牡蛎(Oyster), 法半夏(Prepared Pinellia Tuber), 炒决明子(Parched Semen Cassiae), 柏子仁(seed of Chinese Arborvitae), 丹参(root of Ligulilobe sage), 大黄(root and rhziome of Sorrel Rhubarb), 麸炒枳实(Fry the bran and bitter orange), 黄芪(root of Membranous Milkvetch), 太子参(Heterophylly Falsestarwort root), 莱菔子(Radish seed), 苦杏仁(Armeniacae Semen Amarum), 制何首乌(Polygonum Multiflorum), 白芍(White peony root), 檀香(Sandalwood), 龙骨(Dragon's bone), 茯苓(Indian Bread), 珍珠母(Nacre), 酸枣仁(Spine Date Seed), 五味子(Schisandrae Chinensis Fructus), 炒苏子(Prepared Purple Perilla fruit), 麦冬(Liriope Equivalent plant), 制吴茱萸(Medicil Evodia fruit).

Table S2 A diagnosis and treatment example given by doctors and PrescDRL.

Type	Treatment number	Symptom of patients	TP	Prescription	Symptom score
Doctor	1	胸憋闷,四肢麻木,舌苔厚,饥饿,皮肤薄,头晕,舌苔黄,自汗,大便干,乏力,下肢水肿,脉沉,皮肤黑,口干渴,全身发冷,脉象细,头胀	63	麸炒枳实,白芍,砂仁,酸枣仁,丹参,柏子仁,苍术,藿香,龙骨,檀香,牡蛎,苦参,法半夏,黄芩,厚朴	17
	2	胸憋闷,四肢麻木,呕吐泛酸,舌苔薄,舌苔胖大,小便多,心悸,手颤动,胁胀,舌苔黄,小便尿频,恶寒,大便干,食少,全身发冷,脉弦,胃脘不适	63	麸炒枳实,白芍,砂仁,丹参,地龙,柏子仁,五味子,龙骨,杜仲,檀香,牡蛎,太子参,珍珠母,大黄,麦冬,法半夏	17
	3	胸憋闷,头晕,舌苔黄,自汗,全身乏力,脉沉,心慌,全身发冷,脉细	93	麸炒枳实,黄芪,白芍,砂仁,丹参,苍术,红花,檀香,牛膝,大黄,川芎,当归,法半夏,厚朴,桂枝	9
PrescDRL	1	胸憋闷,四肢麻木,舌苔厚,饥饿,皮肤薄,头晕,舌苔黄,自汗,大便干,乏力,下肢水肿,脉沉,皮肤黑,口干渴,全身发冷,脉象细,头胀	63	山茱萸,牡丹皮,砂仁,菟丝子,丹参,覆盆子,杜仲,益智仁,檀香,大黄,甘草,生地黄,茯苓,鹿角霜,熟地黄,泽泻	17
	2	胸憋闷,干咳,下肢乏力,胸痛,手足麻木,头晕,目昏,舌苔黄,大便干,脉细,脉弦,有痰,头胀	63	麸炒枳实,白芍,陈皮,丹参,地龙,竹茹,龙骨,杜仲,牡蛎,珍珠母,大黄,甘草,川芎,当归,法半夏	13
	3	胸憋闷,干咳,手足麻木,头晕,目昏,舌苔黄,大便干,心慌,脉细,脉弦,有痰,头胀	93	天麻,麸炒枳实,瓜蒌,酸枣仁,柏子仁,五味子,竹茹,太子参,薤白,麦冬,白术,法半夏	12

- TP denotes the treatment plan.
- The English translations for the symptom names mentioned in the case are as follows: 舌苔厚(thick coated tongue), 心慌(palpitation), 目昏(blurred vision), 小便尿频(frequent urination), 全身乏力(malaise), 胸憋闷(chest suffocated), 脉沉(deep pulse), 恶寒(aversion to cold), 大便干(dry stool), 自汗(spontaneous sweating), 干咳(dry cough), 乏力(fatigue), 舌苔胖大(fat tongue coating), 胸痛(chest pain), 头胀(fullness in head), 舌苔薄(thin coated tongue), 舌苔黄(yellow tongue coating), 脉弦(pulse string), 饥饿(hunger), 脉象细(thin pulse), 有痰(with phlegm), 食少(reduced appetite), 皮肤薄(thin skin), 全身发冷(chill all over), 心悸(palpitation), 胁胀(rib-side distention), 脉细(thin pulse), 呕吐泛酸(pantothenic acid vomiting), 小便多(frequent urination), 胃脘不适(Epigastric discomfort), 头晕(dizziness), 皮肤黑(black skin), 下肢水肿(edema lower extremity), 手颤动(hand tremor), 手足麻木(hand and foot numbness), 口干渴(thirsty mouth), 下肢乏力(lower limb weakness), 四肢麻木(limbs anesthesia).
- The English translations for the Chinese herbal names mentioned in the case are as follows: 厚朴(bark of Officil Magnolia), 黄芪(root of Membranous Milkvetch), 藿香(Agastache Rugosa), 山茱萸(Asiatic Cornelian

Cherry fruit), 珍珠母(Nacre), 五味子(Schisandrae Chinensis Fructus), 益智仁(Sharpleaf Galangal), 熟地黄(Prepared Rehmannia root), 牛膝(root of Twotooth Achyranthes), 檀香(Sandalwood), 丹参(root of Ligulilobe sage), 地龙(Earthworm), 酸枣仁(Spine Date Seed), 鹿角霜(Degelatined Deer-horn), 麦冬(Liriope Equivalent plant), 大黄(root and Rhziome of Sorrel Rhubarb), 柏子仁(seed of Chinese Arborvitae), 砂仁(Villous Amomum fruit), 法半夏(Prepared Pinellia Tuber), 桂枝(Cassia Twig), 生地黄(Fresh Rehmannia root), 天麻(Tall Gastrodia), 薤白(bulb of Longstamen Onion), 麸炒枳实(Fry the bran and bitter orange), 竹茹(Bamboo Shavings), 瓜蒌(Trichosanthes Kirilowii Maxim), 牡蛎(Oyster), 菟丝子(Dodder Seed), 白芍(White peony root), 太子参(Heterophylly Falsestarwort Root), 红花(Safflower), 当归(root of Chinese Angelica), 牡丹皮(Tree Peony Bark), 甘草(Root of Ural Licorice), 陈皮(Dried Tangerine Peel), 川芎(Chuanxiong Rhizome), 茯苓(Indian Bread), 龙骨(Dragon's bone), 苦参(root of Lightyellow Sophora), 泽泻(rhizome of Oriental Waterplantain), 覆盆子(Rubi Fructus), 黄芩(root of Baikal skullcap), 白术(Rhizome of Largehead Atractylodes), 杜仲(Eucommia Bark), 苍术(Rhizome of Swordlike Atractylodes)

Table S3 A diagnosis and treatment example given by doctors and PrescDRL.

Type	Treatment number	Symptom of patients	TP	Prescription	Symptom score
Doctor	1	舌苔厚腐腻,舌苔胖大,目昏,大便干,小便夜尿,脉沉,脉弦,舌苔红	10	鸡血藤,干姜,香附,清半夏,黄连,当归,柴胡,黄芩	8
	2	目干,全身疼痛,大便干,小便夜尿,饮食不渴	16	鸡血藤,续断,炒杜仲,干姜,香附,清半夏,大黄,黄连,当归,柴胡,黄芩	5
	3	胸憋闷,盗汗,舌苔薄,失眠,头晕,舌苔黄,全身乏力,脉沉,脉细,头痛	20	黄柏,煅龙骨,黄芪,酸枣仁,五味子,干姜,大黄,知母,浮小麦,黄连,当归,黄芩,煅牡蛎	10
	4	目胀,舌苔黄,健忘,心烦,全身发冷	25	黄柏,黄芪,鸡血藤,干姜,黄连,当归,生地黄,茯苓,黄芩,桂枝,泽泻	5
PrescDRL	1	舌苔厚腐腻,舌苔胖大,目昏,大便干,小便夜尿,脉沉,脉弦,舌苔红	10	鸡血藤,酸枣仁,干姜,香附,清半夏,大黄,黄连,当归,柴胡,黄芩	8
	2	目干,目昏,大便干,全身麻木	78	葛根,天花粉,干姜,片姜黄,桑枝,黄连,松节,桂枝	4
	3	手麻木,舌苔胖大,目昏,大便干	76	天花粉,干姜,桑枝,白术,黄连,茯苓,黄芩,桂枝,党参	4

- TP denotes the treatment plan.
- The English translations for the symptom names mentioned in the case are as follows: 胸憋闷(chest suffocated), 目胀(eye bilges), 全身乏力(malaise), 全身疼痛(generalized pain), 目昏(blurred vision), 饮食不渴(diet not thirsty), 全身发冷(chill all over), 脉细(thin pulse), 脉沉(deep pulse), 手麻木(hand numbness), 舌苔厚腐腻(thick and greasy tongue coating), 健忘(amnesia), 脉弦(pulse string), 小便夜尿(urination nocturia), 舌苔红(red tongue coating), 头晕(dizzy), 心烦(dysphoria), 舌苔黄(yellow tongue coating), 盗汗(night sweat), 舌苔胖大(fat tongue coating), 头痛(headache), 大便干(dry stool), 舌苔薄(thin coated tongue), 目干(stem of eye), 全身麻木(general numbness), 失眠(insomnia).
- The English translations for the Chinese herbal names mentioned in the case are as follows: 葛根(root of lobed Kudzuvine), 桑枝(Mulberry Twig), 泽泻(rhizome of Oriental Waterplantain), 松节(Loose Knot), 黄连(Rhizome of Chinese Goldthread), 黄芩鸡血藤(Spatholoba scutellaria), 生地黄(Fresh rehmannia root), 五味子(Schisandrae Chinensis Fructus), 炒杜仲(Fried Eucommia), 当归(root of Chinese Angelica), 酸枣仁(Spine Date Seed), 干姜(Dried Ginger), 清半夏(Alum Processed Pinellia), 白术(Rhizome of Largehead Atractylodes), 黄芩(root of Baikal Skullcap), 知母(rhizome of Common Amarrhe), 柴胡(root of Chinese Thorowax), 党参(root Pilose Asiabell), 桂枝(Cassia Twig), 茯苓(Indian Bread), 煅龙骨(Prepared Dragon's bone), 香附(rhizome of Nutgrass Galingale), 大黄(root and Rhziome of Sorrel Rhubarb), 浮小麦(Light wheat), 煅牡蛎(Calcined oyster shell), 黄柏(Phellodendron Bark), 片姜黄(Wenyujin Concise Rhizome), 天花粉(Radices Trichosanthis), 鸡血藤(Suberect Spatholobus Stem), 续断(Himalayan Teasel root), 黄芪(root of Membranous Milkvetch).
